# Upregulation of *FOXO3* in New-Onset Type 1 Diabetes Mellitus

**DOI:** 10.1155/2020/9484015

**Published:** 2020-08-12

**Authors:** Magdalena Zurawek, Marta Fichna, Piotr Fichna, Maria Czainska, Natalia Rozwadowska

**Affiliations:** ^1^Institute of Human Genetics, Polish Academy of Sciences, Poznan, Poland; ^2^Department of Endocrinology, Metabolism and Internal Medicine, Poznan University of Medical Sciences, Poznan, Poland; ^3^Department of Paediatric Diabetes and Obesity, Poznan University of Medical Sciences, Poznan, Poland; ^4^Family Physician Clinic, Murowana Goslina, Poland

## Abstract

Forkhead box O (FOXO) transcription factors have been implicated in the development and differentiation of the immune cells. FOXO3 plays a crucial role in physiologic and pathologic immune response. FOXO3, cooperatively with FOXO1, control the development and function of Foxp3^+^ regulatory T cells (T_reg_). Since the lack of T_reg_-mediated control has fundamental impact on type 1 diabetes mellitus (T1DM) development, we investigated *FOXO3* expression in patients with T1DM. *FOXO3* expression was estimated in peripheral blood mononuclear cells (PBMCs) from newly diagnosed T1DM pediatric patients (*n* = 28) and age-matched healthy donors (*n* = 27) by reahavel-time PCR and TaqMan gene expression assays. Expression analysis revealed significant upregulation of *FOXO3* in T1DM (*P* = 0.0005). Stratification of the T1DM group according to the presence of initial diabetic ketoacidosis (DKA) did not indicate differences in *FOXO3* expression in patients with DKA compared to a mild T1DM onset (*P* > 0.05). In conclusion, overexpression of *FOXO3* is correlated with the ongoing islet autoimmune destruction and might suggest a potential role for this gene in the pathogenesis of type 1 diabetes mellitus.

## 1. Introduction

FOXO3 (forkhead box O3) protein belongs to the family of transcription factors included withal FOXO1, FOXO4, and FOXO6. FOXO3 is regulated *via* the phosphoinositide 3-kinase (PI3K)/serine/threonine-specific kinase (Akt) signaling pathway [1]. The active, nonphosphorylated FOXO3 form is localized in the nucleus and regulates gene transcription. Phosphorylation of FOXO3 in the PI3K/Akt pathway results in its exclusion from the nucleus and termination of transcriptional activity [2]. FOXO3 has been implicated in the regulation of diverse biological processes, including cell survival, proliferation, and apoptosis [3]. *FOXO3* is expressed in immune cells, and recently, there has been a surge in interest to investigate the importance of FOXO3 in lymphoid homeostasis [4–6]. Upregulation of FOXO3 was observed in polymorphonuclear cells and peripheral blood mononuclear cells from patients with rheumatoid arthritis [7]. Overexpression of FOXO3 is mediated by T cell receptor stimulation [8]. In consequence, FOXO3 promotes polarization of CD4^+^ T cells towards the pathogenic T helper cells producing interferon *γ* and granulocyte monocyte colony-stimulating factor. *FOXO3*^−^/^−^ mice exhibit reduced susceptibility to experimental autoimmune encephalomyelitis [8].

In this study, we investigate the expression level of *FOXO3* in PBMCs from newly diagnosed type 1 diabetes mellitus pediatric patients. Upregulation of *FOXO3* was observed in the T1DM group compared to the age-matched healthy controls—a finding that might suggest a potential role of this gene in autoimmunity.

## 2. Study Groups

The qRT-PCR of *FOXO3* gene was conducted in 28 newly diagnosed T1DM subjects (mean age ± SD 11.2 ± 3.3 years, 4 females (14%), 24 males (86%)) and 27 age-matched healthy donors (mean age ± SD 10.8 ± 3.9 years, 13 (48%) females, 14 (52%) males). Patients were recruited at the Department of Paediatric Diabetes and Obesity, Poznan University of Medical Sciences. The diagnosis of diabetes was based upon the WHO criteria. Autoimmune origin of the disease was confirmed by positive serum autoantibodies to insulin (IAA) and/or glutamic acid decarboxylase (GADA) and/or islet antigen-2 (IA-2A). Clinical characterization of patients is summarized in Table 1. Pediatric control individuals with negative personal and family history of autoimmunity and no clinical signs of the autoimmune disorders were obtained from an outpatient pediatric practice in the course of routine screening. Only subjects with no clinical symptoms of hyperglycemia and fasting blood glucose within the reference range (≤5.5 mmol/l) were included in the control group.

## 3. Methods

### 3.1. *FOXO3* Expression Analysis

*FOXO3* expression was assessed in PBMCs isolated from the peripheral blood (4 ml for each subject) by density gradient centrifugation in Histopaque-1077 (Sigma Aldrich, Germany). Total RNA was extracted with a TRI Reagent (Sigma Aldrich, Germany) following the manufacturer's protocol. The equal amount of RNA (500 ng per sample) was converted to cDNA using a QuantiTect Reverse Transcription Kit (QIAGEN, Germany). A quantitative real-time PCR using an aliquot of cDNA equivalent of 5 ng total RNA, TaqMan gene expression assay (Applied Biosystems, Thermo Fisher Scientific, USA), and HOT FIREPol Probe Universal qPCR Mix (Solis BioDyne, Estonia) was performed in a total volume of 15 *μ*l on a BioRad CFX96 Real-Time PCR instrument (BioRad Laboratories, CA, USA). All reactions were run in triplicate. The expression level of *FOXO3* (assay ID Hs00818121_m1) was normalized to *beta-2 microglobulin* housekeeping gene (assay ID Hs00984230_m1). Mean cycle threshold (Ct) values were estimated with BioRad CFX Manager 3.1 software. Relative expression levels were calculated using the 2^−*ΔC*t^ formula. Statistical analysis was performed using GraphPad Prism 5 (GraphPad Software Inc., CA, USA). Statistical significance of the differences between relative expression levels was determined with an unpaired *t*-test. *P* values < 0.05 were considered statistically significant.

## 4. Results and Discussion

Type 1 diabetes mellitus is an autoimmune disorder that results from the lack of endogenous insulin secretion from the pancreatic beta cells. Although T-mediated destruction of beta cells is observed, the precise etiology and pathological mechanisms are still poorly understood. Genetic predisposition and environmental factors contribute to the development of type 1 diabetes mellitus [9]. HLA locus, specifically the haplotypes DRB1∗03-DQA1∗05-DQB1∗02 (DR3-DQ2) and DRB1∗04-DQA1∗03-DQB1∗03:02 (DR4-DQ8) are major genetic risk factors [10]. To date, around 60 non-HLA T1DM susceptibility loci have been identified, mostly related to immune response, for instance, genes encoding lymphocyte protein tyrosine phosphatase (*PTPN22*), cytotoxic T-lymphocyte protein 4 (*CTLA4*), subunit alpha of the interleukin-2 receptor (*IL2RA*), and interferon-induced helicase C domain-containing protein 1 (*IFIH1*) [11–13].

**Figure 1:**
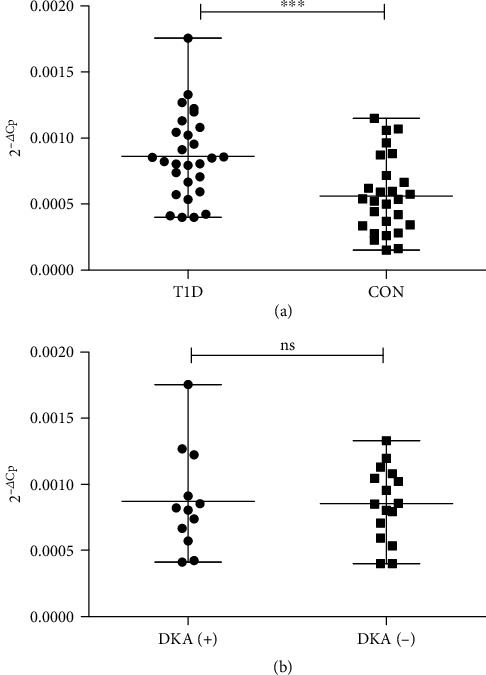
*FOXO3* expression analysis in type 1 diabetes. (a) *FOXO3* is overexpressed in the type 1 diabetes group compared to healthy controls (^∗∗∗^*P* = 0.0005, mean fold change 1.54). (b) Expression level of *FOXO3* gene in the T1D group stratified according to the presence of initial diabetic ketoacidosis (DKA). The patients with diabetic ketoacidosis did not present statistically significant differences in *FOXO3* expression compared to the patients without DKA (*P* > 0.05). Horizontal lines indicate median with range; asterisks indicate significance, with *P* values estimated by unpaired *t*-test; T1D: type 1 diabetes patients; C: controls; DKA(+): cohort of patients with initial diabetic ketoacidosis; DKA(-): cohort of patients without initial diabetic ketoacidosis.

Our previous study indicated *FOXO3* as a potential target for miR-487a-3p, which is upregulated in T1DM [14]. These results prompted us to investigate the *FOXO3* expression in type 1 diabetes mellitus patients. In order to reduce the interference of the initial metabolic status, PBMCs were collected from patients with normalized ketonaemia and glycemia and fully rehydrated. In addition, T1DM patients and control subjects included in the study did not present infection symptoms, confirmed by negative inflammatory tests (complete blood count, C-reactive protein tests). The type 1 diabetes mellitus group was further stratified according to the presence or absence of diabetic ketoacidosis (DKA) at initial presentation, which reflects severe and moderate disease onsets, respectively. Expression analysis revealed significant upregulation of *FOXO3* in the new-onset T1DM group compared to the age-matched healthy controls (Figure 1). However, we did not observe statistically significant differences in *FOXO3* expression in patients with DKA compared to the mild T1DM onset group (Figure 1). Consequently, *FOXO3* expression is correlated with the ongoing autoimmune islet destruction, although not with the severity of the autoimmune process. A current study failed to confirm the previous global expression analysis of PBMCs from children with newly diagnosed type 1 diabetes mellitus (GEO DataSets, Accession GDS3875) [15]. The microarray analysis did not reveal dysregulation of *FOXO3* in the group of T1DM patients at one month and at four months after diagnosis [15].

Based on our previous study, we hypothesized that overexpression of miR-487a-3p in T1DM might result in downregulation of *FOXO3* and hence affect the immune function. Current results imply that *FOXO3* is apparently not regulated *via* miR-487a-3p; however, additional *in vitro* study is needed to explore the manner of *FOXO3* regulation. Yang et al. have demonstrated the mechanisms protecting *FOXO3* from being targeted by certain miRNAs [16]. The *Foxo3* pseudogene (Foxo3P) and the Foxo3 circular RNA (circ-Foxo3) act as a sponge and bind several miRNAs, including miR-22, miR-136, miR-138, miR-149, miR-433, miR-762, miR-3614-5p, and miR-3622b-5p. Subsequently, *Foxo3P* and circ-Foxo3 ensure *FOXO3* gene expression and protein activity.

## 5. Conclusion

Overexpression of *FOXO3* in type 1 diabetes mellitus might suggest a potential role of this gene in the development of autoimmune disease. Further *in vitro* and *ex vivo* functional studies will address the issue of *FOXO3* contribution to immune tolerance dysregulation.

## Figures and Tables

**Table 1:** Clinical characteristics of type 1 diabetes patients.

Clinical features	T1D	Severe T1D onset	Mild T1D onset
	*n* = 28 (%)	*n* = 12 (%)	*n* = 16 (%)
Gender, F/M	4/24		
Age^†^ (y)	11.21 ± 3.33	11.33 ± 3.63	11.63 ± 3.18
BMI^†^ (kg/m^2^)	17.33 ± 3.05	17.31 ± 3.18	17.62 ± 3.09
HbA1c^†^ (%)	10.66 ± 1.67	10.59 ± 1.15	11.16 ± 1.86
C peptide^†^ (nmol/l)	0.30 ± 0.08	0.27 ± 0.9	0.32 ± 0.08
25-OH-D^†^ (ng/ml)	18.19 ± 7.68	13.98 ± 3.27^∗∗^	21.73 ± 8.76
DKA^‡^	12 (43)	12 (100)	16 (0)
IAA^‡^	7 (25)	3 (25)	4 (25)
GADA^‡^	21 (75)	9 (75)	12 (75)
IA2A^‡^	23 (82)	11 (92)	12 (75)

BMI: body mass index; HbA1c: glycated haemoglobin A1c; 25-OH-D: 25-hydroxyvitamin D; DKA: diabetic ketoacidosis; IAA: antibodies to insulin; GADA: antibodies to glutamic acid decarboxylase; IA2A: antibodies to islet antigen-2; clinical features presented as ^†^mean ± standard deviation; ^‡^number of subjects (%); ^∗∗^*P* < 0.01, *P* values estimated by unpaired *t*-test, severe T1D onset *vs*. mild T1D onset subgroup.

## Data Availability

Data presented in the manuscript are available upon request from corresponding author Magdalena Zurawek, magdalena.zurawek@igcz.poznan.pl.
